# Multidimensional Impact of Smog on Respiratory and Ocular Health: A Cross‐Sectional Study With Socio‐Psychological and Public Health Prospective

**DOI:** 10.1002/hsr2.71205

**Published:** 2025-08-27

**Authors:** Muhammad Muneeb Hassan, Iqra Javaid, Farrukh Jamal, Muhammad Ameeq, Muhammad Danish, Alpha kargbo, Ayesha Javed

**Affiliations:** ^1^ The Islamia University Bahawalpur Bahawalpur Pakistan; ^2^ Department of Statistics DHQ hospital Muzaffargarh Muzaffargarh Pakistan; ^3^ Punjab Thalassaemia & Other Genetic Disorders Prevention and Research Institute Bahawalpur Pakistan; ^4^ Department of Statistics, Faculity of Science University of Tabuk Tabuk Saudia Arabia; ^5^ Shahida Islam Nursing College Lodhran Pakistan; ^6^ Department of Physical and Natural Sciences University of The Gambia Serrekunda Gambia

**Keywords:** air quality index, public health policy and SMOG, respiratory health, socio‐psychological effect

## Abstract

**Background and Aims:**

Smog is a serious threat to the environment, creating problems for public health, particularly in the South Punjab, Pakistan. This study aim to assess the prevalence of smog related health issue evaluate psychological impact and explore public health awareness and policy perception.

**Methods:**

A cross‐sectional study of 817 adults (Aged 18≥ year) was conducted in cities of South‐Punjab from October to December 2024. Structured questionnaire, aligned with the STROBE checklist, collected self‐reported data on health outcomes and awareness. Multinomial logistic regression and Wilcoxon rank‐sum tests were performed using SPSS‐26 and R‐Studio 4.3.2.

**Results:**

From 817 participants, 57.8% were male, 65.7% urban, with 47.1% reporting eye irritation (OR = 1.33, 95% CI: 0.692.56, *p* = 0.38) Respiratory conditions included COPD (OR = 0.22, 95% CI: 0.11–0.41, *p* < 0.001), asthma (OR = 0.22, 95% CI: 0.13–0.38, *p* < 0.001), ARI (OR = 0.18, 95% CI: 0.10–0.31, *p* < 0.001), and IHD (OR = 11.13, 95% CI: 6.81–46.35, *p* < 0.001). Urban participants showed higher anxiety due to smog (OR = 6.20, 95% CI: 2.56– 15.04, *p* < 0.001). Only 15.9% were aware of public health campaigns, and 62.7% rated government efforts poorly (OR = 2.69, 95% CI: 1.40–5.17, *p* = 0.003).

**Conclusion:**

Smog significantly affects respiratory and ocular health causes socio‐psychological burdens. Strict regulations, effective public health interventions, and healthcare infrastructure are essential to decrease its effects.

## 1 Introduction

Public health outcomes are greatly affected by air quality, particularly with it respect to respiratory and ocular conditions [1]. The air quality index (AQI) is a standardized measure used to assess and communicate air quality in specific areas, reflecting the concentrations of pollutants, such as particulate matter (PM10, PM2.5), ozone (O3), sulfur dioxide (SO2), carbon monoxide (CO), and nitrogen dioxide (NO2) [2, 3]. Millions of premature deaths occur annually owing to air pollution. The Air Quality Index (AQI) includes different categories that range from good to dangerous. A value greater than 300 indicates severe air quality, while a value less than 50 indicates healthy air quality. The World Health Organization states that in low‐ and middle‐income countries, seven million people die due to air pollution [4, 5, 6].

**Figure A1:**
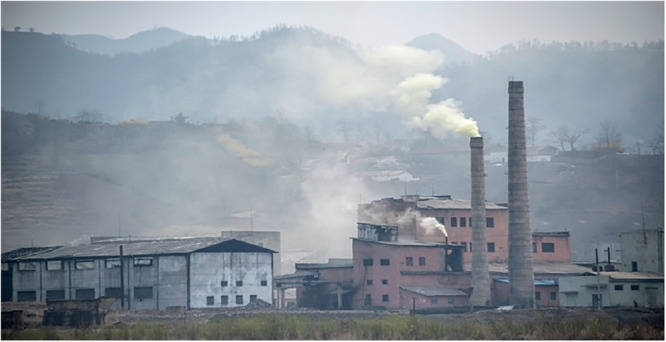
Industrial pollution.

**Figure A2:**
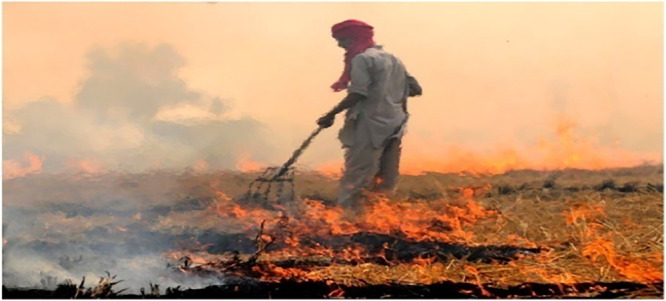
Smog produce due to crops burning.

Every year, Pakistan there is a severe air quality crisis. The ranking is consistently increasing among the world's most polluted countries, including Lahore, Multan, Bahawlpur, and Peshawar, where AQI levels remain high [7]. According to the WHO guideline, the average annual (PM2.5) absorption is (10 ug/m^3^), but in urban areas it regularly exceeds (100 µg/m^3^), which is considerably greater. Regions like Punjab, where smog is heavily caused by industrial activities, automobiles, and agricultural practices, are particularly at risk for the negative effects of air pollution on public health as shown in Figure A1 and Figure A2 [8]. The decline in air quality was most evident in the southern Punjab region, encompassing the districts of Muzaffargarh, Bahawalpur, and Multan. The AQI in these regions frequently attains unhealthy levels owing to multiple factors, including dust from unpaved roads, crop burning during harvest seasons, and emissions from brick kilns. Smog is widespread during the winter months, which causes the major spread of respiratory systems like chronic obstructive pulmonary disease (COPD), asthma, acute respiratory infection (ARI), ischemic heart disease (IHD), and conjunctivitis. South Punjab healthcare units reports alarming trends in this health condition [9, 10, 11].

When the AQI level increases above 200, patients with COPD are at a high risk, and asthmatic patients experience the most severe attacks. In smog, air particles irritate the eyes, leading to an increase in the incidence of conjunctivitis. It has been observed that ARI patients increased during smog sessions compared to non‐smog periods [12, 13, 14, 15].

A comprehensive approach that considered both the socio‐psychological effects on impacted populations as well as physical health outcomes is necessary to comprehend the multidimensional impact of smog on health. In the South Punjab, where air pollution is a chronic problem, many people experience health‐related anxiety and stress. This negative impact on mental health can worsen existing health conditions and lower quality of life [16, 17]. This study explores the complex relationship between air pollution and its impact on eye and respiratory health in the southern Punjab region with a particular emphasis on the districts of Muzaffargarh, Bahawalpur and Multan [18, 19, 20]. This study examined self reported cases of ARI, asthma, COPD, IHD, conjunctivitis case data, and AQI measurements to provide new information about the impact of air pollution on public health [21, 22, 23]. It highlights the need for effective public health interventions that reduce the effects of poor air quality on these most susceptible. This requires coordinated efforts from local community emission reduction efforts to national environmental regulation reforms. This shows the need for stronger public health responses to smog.

Urban air pollution has emerged as a critical environmental and health concern across South Asia, with rapid urbanization, industrialization, and vehicular emissions contributing significantly to deteriorating air quality. Several recent studies have emphasized the growing burden of smog and its health implications in this region. Influence of seasonal variability and urban green spaces on ambient air quality in Pakistan, highlighting the role of local environmental factors in pollution levels [24]. Geo‐visualized smog‐induced health effects in Lahore, offering a community‐based perspective on exposure and vulnerability [25]. Global attention to address Lahore's worsening air pollution, linking it directly to health deterioration and policy inaction [26, 27]. These studies support the rationale and urgency of our research, particularly in contextualizing the multidimensional impacts of smog within South Asia's densely populated and pollution‐prone urban environments.

It tests three hypotheses: (H1) Smog exposure is associated with adverse respiratory health outcomes; (H2) Smog exposure is associated with adverse ocular health outcomes; (H3) Smog exposure significantly affects psychological well‐being.

### 1.1 Aims and Objectives

This study aims to evaluate smogs multidimensional impact on respiratory, ocular, and socio‐psychological health in South Punjab. Specific objectives are: (1) Determine the prevalence of ARI, asthma, COPD, IHD, and conjunctivitis; (2) Assess socio‐psychological effects (stress, anxiety); (3) Evaluate public health and policy awareness; (4) Analyze the effectiveness of protective measures.

## 2 Materials and Methods

### 2.1 Study Design and Setting

**Figure 1:**
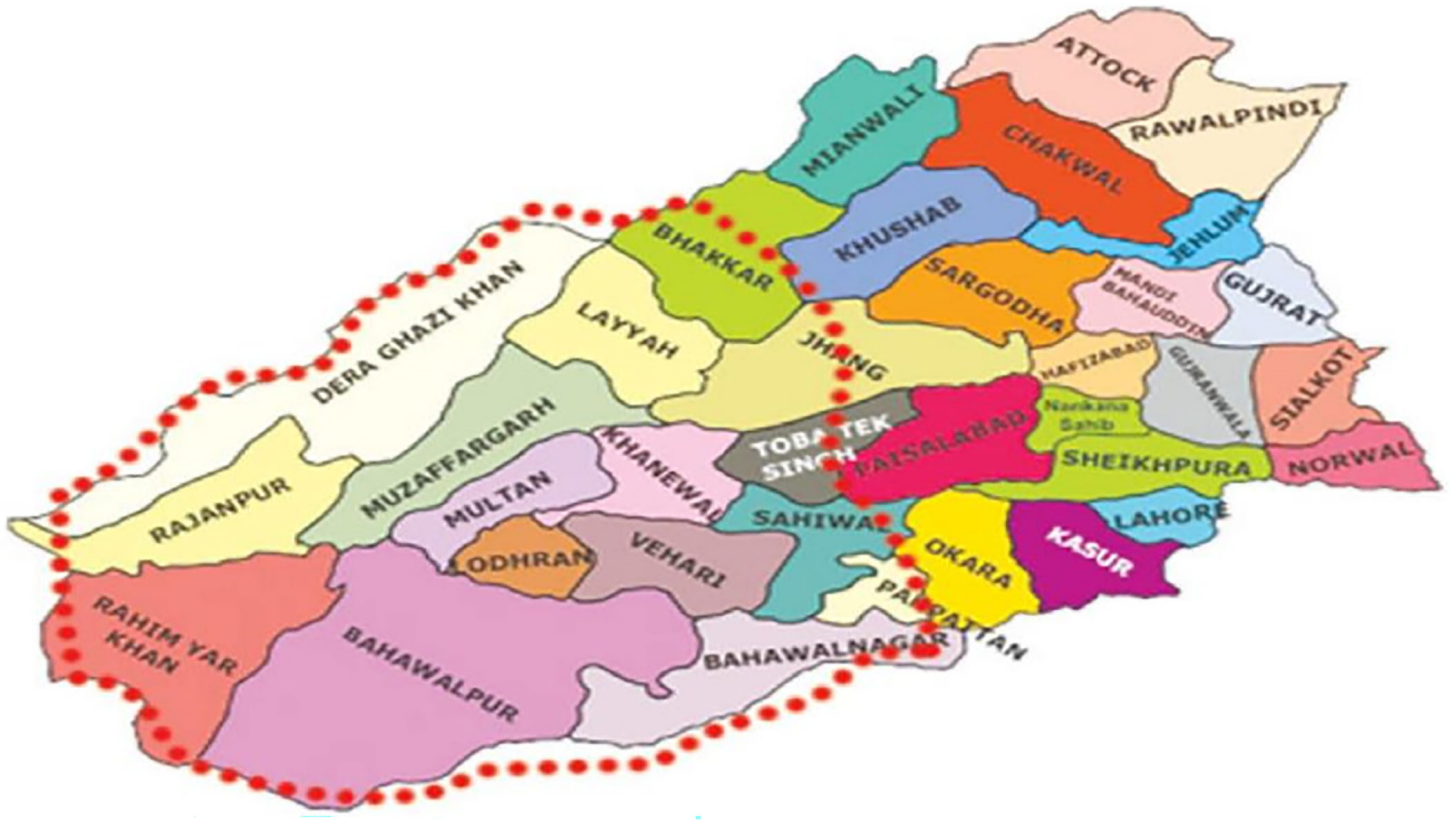
Map of southern‐Punjab region in Pakistan.

This cross‐sectional study examined the Multidimensional impact of smog on respiratory and ocular health using socio‐psychological and public health dimensions. This study was conducted in the Southern Punjab region, Pakistan which focuses on different district regions Multan, Bahawalpur such as, Muzaffargarh as shown in Figure 1.

The process of gathering data began in the winter months, time marked by elevated smog levels resulting from climatic influences, industrial discharges, and, agricultural circumstances. We examined both urban and rural populations to gain a comprehensive understanding of exposure and associated health outcomes. Data were collected from October to November 2024, finalizing data entry, coding, analysis, and report composition, and concluding the study in Dec 2024. Average AQI levels in October (234), November (262), and December (271) exceeded the national threshold (100), indicating hazardous conditions.

### 2.2 Study Population and Sampling

This study, patients who were 18 years and older and who had lived in their current place for a minimum of 1 year were included, ensuring they had experience of local environmental conditions. We used stratified random sampling techniques to ensure effective representation of both rural and urban populations and the sample size was calculated using Cochran's formula, incorporating a 95% confidence interval and 5% margin of error. Patients were recruited from healthcare facilities, residential communities, and adjacent organizations (educational institutes, commercial centers, and NGOs) to ensure diverse representation of the sample.

### 2.3 Ethical Approval and Informed Consent

Ethical approval was obtained from the Institutional Review Board of DHQ Hospital Muzaffargarh No, 1003‐7/DHQ dated: 01 Jan 2025. All participants provided informed verbal consent, approved by the DHQ Hospital Muzaffargarh Institutional Review Board, in accordance with the Declaration of Helsinki, after being fully informed about the study's objectives, procedures, risks, benefits, confidentiality, and their right to withdraw without consequences, with thumb impressions used as documentation due to high illiteracy rates.

### 2.4 Data Collection Tools and Procedures

We collected primary data from a structured questionnaire to strengthen the reporting of observational epidemiological studies using the STROBE Checklist. The questionnaire included items assessing household air pollution sources, including indoor smoking and biomass fuel usage. This study included six sections regarding age, gender, education level, occupation, area (rural or urban), and how long they had lived in their community. Urban patients reported more frequent exposure to vehicular and industrial emissions, while rural participants were more exposed to biomass and agricultural pollutants. Information regarding respiratory and ocular health was collected to categorize participants based on their symptom severity. The patient's information focused on whether they experienced eye discomfort due to smog exposure in a polluted environment during their visit to the eye specialist. The psychological effects of pollution were evaluated by measuring levels of stress and anxiety in patients. This study examined how smog affectes everyday activities. We classified the patients as either urban or rural based on their respiratory symptoms and where they lived. To evaluate public opinion on the smog campaign and the city's initiatives to reduce air pollution, we pooled our patients. The survey inquired about local smog source and patients' views on community air pollution.

### 2.5 Statistical Analysis

Anxiety and stress levels, as well as other measures of psychological health, were compared between rural and urban patients using the Wilcoxon rank‐sum test. The impact of smog exposure on health outcomes, particularly ocular and respiratory health, was investigated using multinomial logistic regression. Statistical analyses were conducted using SPSS‐26 and R version 4.3.2.

### 2.6 Hypotheses

People with higher smog exposure reported much more stress and anxiety than those with lower exposure. Mild to severe respiratory problems are associated with smog exposure. More severe ocular symptoms were associated with higher smog exposure. According to our study objectives: there are three hypotheses:

Smog exposure is associated with adverse respiratory health outcomes.

Smog exposure is associated with adverse ocular health outcomes.

Smog exposure significantly affects psychological well‐being.

## 3 Results

Descriptive Table 1 highlights the socio‐psychological and public health perspectives as well as the multidimensional impact on respiratory and ocular health. The study involved 817 patients, of whom 57.8% were male and 49% were aged between 18 and 30 years. The urban population consists of 65.7%, which is highly affected by smog. Of the total population has, 43.8% had completed primary education, while 10.4% had completed college or university education. After smog exposure, 47.1% reported symptoms such as irritation in the eyes. A total of 8.6% of people wore eyewear protection, highlighting the lack of preventive measures. Nearly 45.4% used respiratory medication, and 33.7% were diagnosed with lung conditions affected by air pollution. In the respiratory health category, a significant proportion, (73.3%), reported experiencing shortness of breath, whereas 58.5% reported experiencing coughing and wheezing.

**Table 1:** Descriptive table of multidimensional impact of smog‐affected patients on respiratory and ocular health: a socio‐psychological and public health perspective.

Characteristics	Total (%)	Characteristics	Total (%)
**Gender** Male Female **Total**	— 472 (57.8) 345 (42.2) 817	**Ocular health**	
**Age** 18–30 31–50 51–70 and above	— 400 (49) 274 (33.5) 143 (15.5)	Have you experienced eye irritation (itchiness, redness, watering, etc.) during or after exposure to smog? Yes No	— — — 385 (47.1) 432 (52.9)
**Education** No education Primary school Secondary school College/University	— 247 (30.2) 358 (43.8) 127 (15.5) 85 (10.4)	How frequently do you experience eye dryness or discomfort in smoggy conditions? Never Occasionally Often Always	— — 396 (48.5) 120 (14.7) 225 (27.5) 76 (9.3)
**Area** Rural Urban	— 280 (34.3) 537 (65.7)	Do you wear protective eyewear when exposed to smog? Yes No	— — 70 (8.6) 747 (91.4)
**Diseases** COPD Asthma ARI IHD Conjunctivitis	— 127 (15.5) 223 (27.3) 210 (25.7) 157 (19.2) 100 (12.2)	Have you noticed a change in your vision (e.g., blurred vision) during or after smog exposure? Yes No	— — 193 (23.6) 624 (76.4)
**Respiratory condition**		Have you consulted an eye specialist due to smog‐related eye issues? Yes No	— — 180 (22) 637 (78)
**In past, experienced coughing or wheezing?** Never Occasionally Often Always	— 92 (11.3) 215 (26.3) 478 (58.5) 32 (3.9)	Socio‐psychological impact	
**Have you noticed increased shortness of breath or difficulty breathing in the presence of smog?** Yes No	— — 599 (73.3) 218 (26.7)	On a scale of 1–5, how worried are you about the long‐term health effects of smog? Not worried Slightly worried Moderately worried Very worried Extremely worried	— — 68 (8.3) 48 (5.9) 215 (26.3) 351 (43) 135 (16.5)
**In the last year, how many times have you visited a healthcare professional for respiratory problems?** Never 1–2 time 3–5 Time More than 5 time	— — — 227 (27.8) 237 (29) 270 (33) 83 (10.2)	How often do you feel stressed or anxious due to smog in your area? Never Occasionally Always Often	— — 90 (11) 169 (20.7) 500 (61.2) 58 (7.1)
**Have you ever been diagnosed with a lung condition aggravated by air pollution?** Yes No	— — 275 (33.7) 542 (66.3)	Have you experienced any changes in your mental well‐being (e.g., anxiety, depression) related to air pollution exposure? Yes No	— — — 486 (59.5) 331 (40.5)
**Do you use any respiratory medications (e.g., inhalers, nebulizers)?** Yes No	— — 371 (45.4) 446 (54.6)	Do you feel that smog has negatively affected your quality of life? Yes No	— — 766 (93.8) 51 (6.2)
**Public health prospective**		How has smog affected your daily activities (e.g., outdoor exercise, commuting)? Not at all A little Allot	— — 39 (4.8) 485 (59.4) 293 (35.9)
**Are you aware of any public health campaigns or initiatives addressing smog in your area?** Yes No	— — 130 (15.9) 687 (84.1)	Environmental and public policy awareness	
**Do you believe that air quality monitoring is sufficient in your region?** Yes No	— — 150 (18.4) 667 (81.6)	Are you familiar with the sources of smog in your region? Yes No	— — 454 (55.6) 363 (44.6)
**How would you rate the local government's efforts to reduce air pollution?** Poor Fair Good	— — 512 (62.7) 209 (25.6) 96 (11.8)	Do you support policies aimed at reducing air pollution (e.g., stricter vehicle emissions standards, industrial regulations)? Yes No	— — — 753 (92.2) 64 (7.8)
**Are you aware of any protective measures (e.g., wearing masks, avoiding outdoor activities) recommended during smog?** Yes No	— — — 383 (46.9) 434 (53.1)	How important do you think it is to address air pollution on a community level? Not important Somewhat important Very important	— — 12 (1.5) 277 (33.9) 528 (64.6)
**How often do you follow recommended safety measures during smog episodes?** Never Sometime Often Always	— — 305 (37.3) 295 (36.1) 167 (20.4) 50 (6.1)	General health perception	
		How would you rate your general health? Poor Fair Good Excellent	— 209 (25.6) 213 (26.1) 372 (45.5) 23 (2.8)
		Have you noticed any worsening of chronic conditions (e.g., asthma, heart disease) due to smog exposure? **Yes** **No**	— — 399 (48.8) 418 (51.2)
		How often do you feel fatigued or unwell due to air pollution? Never Occasionally Often Always	— — 56 (6.9) 337 (41.2) 354 (43.2) 70 (8.6)

In light of the socio‐psychological impact, 59.5% felt anxiety or depression in situations that are linked to air pollution. Most participants, 93.8%, believed that smog negatively affected their quality of life; on the other hand, 61.2% felt smog‐stressed. In the public health section, only 15.9% were aware of it, but 92.2% give comments that they needed to make a policy to reduce air pollution. Mostly, 62.7% of people say that there are no proper efforts by local governments to minimize the effect of smog. Only 46.9% of the respondents were familiar with the policy of wearing masks as a protective measure against smog. In terms of general health perception, 48.8% of people believe that smog worsen their chronic condition. Fatigue in the patients was the common factor in which 43.2% were unwell and 41.2% occasionally.

Table 2 displays findings from a multinomial logistic regression analysis investigating the association between multiple factors and the probability of respiratory diseases during smog events. The analysis concentrates on four distinct conditions: Chronic obstructive pulmonary disease (COPD), asthma, acute respiratory infection (ARI), and ischaemic heart disease (IHD). Every row shows a factor or covariate, which includes *p* values, odds ratios (OR), confidence intervals (CI), and coefficients for each condition. Chronic Obstructive Pulmonary Disease (COPD) is marked by notable symptoms like persistent coughing or wheezing. The coefficient stands at −1.48 (SE = 0.26), accompanied by an odds ratio of 0.22 (95% CI: 0.13–0.38), and a p‐value of 0.001. This indicates that increased shortness of breath in smog conditions is a significant determinant. The coefficient is −0.65 (SE = 0.35), and (OR = 0.51; 95% CI: 0.25–1.04; *p* value: 0.065), indicating significant negative correlations and noteworthy p‐values. The administration of respiratory medications significantly decreases the likelihood of COPD. Similar trends to those observed in COPD are apparent, chronic coughing or wheezing and dyspnoea in polluted air serve as significant indicators. A diagnosis of a lung condition with a Coefficient of 1.15 (SE = 0.62), an (OR = 3.17; 95% CI: 0.93–10.96; *p*‐value = 0.065), exacerbated by air pollution, it indicates an exceeding 1, though it does not achieve statistical significance (*p* = 0.065). Acute respiratory infection (ARI) is significantly influenced by factors such as coughing or wheezing (Coefficient = −1.69 (SE = 0.27), (OR = 0.18; 95% CI: 0.10–0.31; *p*‐value = 0.001) and shortness of breath (Coefficient = −4.76 (SE = 0.52), (OR = 0.01; 95% CI: 0.01–0.02; *p*‐value = 0.001) during smog events.

**Table 2:** Multinomial logistic regression analysis of respiratory diseases during smog events.

Factors/covariates^a^, ^b^	OR (95% CI)	*p* value	
**Chronic obstructive pulmonary disease**			
In the past month, how often have you experienced coughing or wheezing?	0.22 (0.11–0.41)	0.001	
Have you noticed increased shortness of breath or difficulty breathing in the presence of smog?	0.01 (0.01–0.02)	0.001	
In the last year, how many times have you visited a healthcare professional for respiratory problems?	0.25 (0.11–0.54)	0.001	
Have you ever been diagnosed with a lung condition aggravated by air pollution?	0.46 (0.12–1.66)	0.237	
Do you use any respiratory medications (e.g., inhalers, nebulizers)?	0.01 (0.01–0.04)	0.001	
**Asthma**		
In the past month, how often have you experienced coughing or wheezing?	0.22 (0.13–0.38)	0.001	
Have you noticed increased shortness of breath or difficulty breathing in the presence of smog?	0.05 (0.02–0.16)	0.001	
In the last year, how many times have you visited a healthcare professional for respiratory problems?	0.51 (0.25–1.04)	0.065	
Have you ever been diagnosed with a lung condition aggravated by air pollution?	3.17 (0.93–10.96)	0.065	
Do you use any respiratory medications (e.g., inhalers, nebulizers)?	0.02 (0.01–0.08)	0.001	
**Acute respiratory infection**			
In the past month, how often have you experienced coughing or wheezing?	0.18 (0.10–0.31)	0.001	
Have you noticed increased shortness of breath or difficulty breathing in the presence of smog?	0.01 (0.01–0.02)	0.001	
In the last year, how many times have you visited a healthcare professional for respiratory problems?	0.35 (0.17–0.72)	0.005	
Have you ever been diagnosed with a lung condition aggravated by air pollution?	0.70 (0.20–2.36)	0.567	
Do you use any respiratory medications (e.g., inhalers, nebulizers)?	0.14 (0.04–0.47)	0.001	
**Ischemic heart disease**			
In the past month, how often have you experienced coughing or wheezing?	0.57 (0.28–1.15)	0.120	
Have you noticed increased shortness of breath or difficulty breathing in the presence of smog?	0.01 (0.01–0.05)	0.001	
In the last year, how many times have you visited a healthcare professional for respiratory problems?	12.82 (4.95–30.44)	0.001	
Have you ever been diagnosed with a lung condition aggravated by air pollution?	11.13 (6.81–46.35)	0.001	
Do you use any respiratory medications (e.g., inhalers, nebulizers)?	0.59 (0.23–1.46)	0.036	

Abbreviations: CI = Confidence Interval; OR = Odds Ratio; SE = Standard error.

^a^ The reference category is: conjunctivitis.

^b^ This parameter is set to zero because it is redundant.

*p‐value < 0.05.

Consultations with healthcare professionals exhibited a significant correlation with the occurrence of acute respiratory infections (ARI), indicated by a coefficient of −1.03 (SE = 0.36), an odds ratio (OR = 0.35 95% CI: 0.17–0.72; *p*‐value = 0.005). The coefficient for shortness of breath in smog is −4.14 (SE = 0.64), the (OR = 0.01; CI: 0.01–0.05; *p*‐value = 0.001), signifying a significant correlation with ischemic heart disease (IHD). Respiratory medications demonstrated a protective effect against ischemic heart disease, indicated by a Coefficient of 4.71 (SE = 0.72), and an (OR = 11.13; CI: 6.81–46.35; *p* = 0.001).

Analysis of public health factors and their associations with health outcomes related to smog exposure. This Table 3 focuses on four major health conditions: Chronic Obstructive Pulmonary Disease (COPD), Asthma, Acute Respiratory Infection (ARI), and Ischemic Heart Disease (IHD). Each row outlines specific factors or covariates and, provid coefficients, odds ratios (OR), confidence intervals (CI), and p‐values for each condition.

**Table 3:** Public health factors and their association with smog related health outcomes.

Factors/covariates^a^, ^b^	OR (95% CI)	*p* value
**Chronic obstructive pulmonary disease**		
Are you aware of any public health campaigns or initiatives addressing smog in your area?	0.60 (0.277–1.34)	0.218
Do you believe that air quality monitoring is sufficient in your region?	0.53 (0.18–1.56)	0.251
How would you rate the local government's efforts to reduce air pollution?	2.52 (1.28–4.98)	0.007
Are you aware of any protective measures (e.g., wearing masks, avoiding outdoor activities) recommended during smog?	1.33 (0.69–2.56)	0.380
How often do you follow recommended safety measures during smog episodes?	1.40 (1.00–1.98)	0.050
**Asthma**		
Are you aware of any public health campaigns or initiatives addressing smog in your area?	1.25 (0.57–2.75)	0.575
Do you believe that air quality monitoring is sufficient in your region?	1.18 (0.41–3.43)	0.750
How would you rate the local government's efforts to reduce air pollution?	2.69 (1.40–5.17)	0.003
Are you aware of any protective measures (e.g., wearing masks, avoiding outdoor activities) recommended during smog?	0.39 (0.21–0.71)	0.002
How often do you follow recommended safety measures during smog episodes?	3.26 (2.36–4.50)	0.000
**Acute respiratory infection**		
Are you aware of any public health campaigns or initiatives addressing smog in your area?	2.15 (0.93–4.94)	0.070
Do you believe that air quality monitoring is sufficient in your region?	2.19 (0.73–6.53)	0.159
How would you rate the local government's efforts to reduce air pollution?	3.14 (1.63–6.07)	0.001
Are you aware of any protective measures (e.g., wearing masks, avoiding outdoor activities) recommended during smog?	1.14 (0.63–2.05)	0.660
How often do you follow recommended safety measures during smog episodes?	1.14 (0.83–1.57)	0.398
**Ischemic heart disease**		
Are you aware of any public health campaigns or initiatives addressing smog in your area?	1.08 (0.45–2.58)	0.847
Do you believe that air quality monitoring is sufficient in your region?	0.67 (0.21–2.05)	0.484
How would you rate the local government's efforts to reduce air pollution?	1.19 (0.58–2.47)	0.627
Are you aware of any protective measures (e.g., wearing masks, avoiding outdoor activities) recommended during smog?	0.34 (0.18–0.61)	0.001
How often do you follow recommended safety measures during smog episodes?	0.59 (0.41–0.86)	0.006

Abbreviations: CI = Confidence Interval; OR = Odds Ratio; SE = Standard error.

^a^ The reference category is: conjunctivitis.

^b^ This parameter is set to zero because it is redundant.

*p‐value < 0.05.

Respondents who rated local government efforts to reduce air pollution positively had a significant association with COPD outcomes, with a coefficient of 0.92 (SE = 0.34), (OR = 2.52; 95% CI: 1.28–4.98; *p*‐value = 0.007). This suggests that a better perceived effort may lead to improved health outcomes. Safety measures during smog episodes were marginally significant with a coefficient of 0.34 (SE = 0.17), (OR = 1.40; 95% CI: 1.00–1.98; *p* = 0.050), indicating that adherence to these measures could potentially reduce risk of COPD.

Similar to COPD, respondents who rated government efforts positively showed strong associations with asthma outcomes, reflected by a coefficient of 0.99 (SE = 0.33), (OR = 2.69; 95% CI: 1.40–5.17; *p*‐value = 0.003). Awareness of protective measures significantly decreased asthma risk, as indicated by a coefficient of −0.93 (SE = 0.30), (OR = 0.39; 95% CI: 0.21–0.71; *p*‐value = 0.002). Those who frequently followed recommended safety measures had a strong positive association with asthma outcomes, as demonstrated by a coefficient of 1.18 (SE = 0.165), (OR = 3.26; 95% CI: 2.36–4.50; *p*‐value < 0.001).

A positive perception of government efforts was also significantly associated with ARI outcomes; the coefficient was 1.14 (SE = 0.33), (OR = 3.14; 95% CI: 1.63–6.07; *p*‐value = 0.001). Awareness of public health campaigns showed a trend toward significance with a coefficient of 0.76 (SE = 0.42), (OR = 2.15; 95% CI: 0.93–4.94; *p*‐value = 0.070). Awareness of protective measures significantly reduced IHD risk, as indicated by a coefficient of −0.10 (SE = 0.30), (OR = 0.34; 95% CI: 0.18–0.61; *p*‐value < 0.001). Conversely, not following recommended safety measures was associated with increased risk for IHD; this is reflected in the coefficient of 0.51 (SE = 0.18), (OR = 0.59; 95% CI: 0.41–0.86; *p*‐value = 0.006).

Socio‐psychological impacts and general health perceptions related to smog exposure and their association with various health outcomes is showed in Table 4. Individuals who express greater concern about the long‐term health effects of smog show a significant association with COPD outcomes, indicated by a coefficient of 1.79 (SE = 0.28), (OR = 5.99; 95% CI: 3.42–10.29; *p*‐value = 0.001). This suggests that increased worry is correlated with higher odds of developing COPD. High levels of stress or anxiety related to smog were strongly associated with COPD, as shown by a coefficient of 3.96 (SE = 0.52), (OR = 5.22; 95% CI: 1.41–14.68; *p* = 0.001). Perceptions that smog negatively affects quality of life also correlate significantly with COPD outcomes, with a coefficient of 2.98 (SE = 0.63), (OR = 1.29; 95% CI: 0.54–4.47; *p* = 0.001). Notably, respondents who noticed worsening chronic conditions due to smog exposure show a very strong negative association with COPD outcomes, indicated by a coefficient of −3.14, (OR = 0.04 (95% CI: 0.02–0.09; *p* = 0.001).

**Table 4:** Socio‐psychological impact and general health perception associated with to smog related outcomes.

Factors/covariates^a^, ^b^	OR (95% CI)	*p* value	
**Chronic obstructive pulmonary disease**			
On a scale of 1–5, how worried are you about the long‐term health effects of smog?	5.99 (3.42–10.29)	0.001	
How often do you feel stressed or anxious due to smog in your area?	5.22 (1.41–14.68)	0.001	
Have you experienced any changes in your mental well‐being (e.g., anxiety, depression) related to air pollution exposure?	1.34 (0.54–3.32)	0.526	
Do you feel that smog has negatively affected your quality of life?	1.29 (0.54–4.47)	0.001	
How has smog affected your daily activities (e.g., outdoor exercise, commuting)?	1.473 (0.64–3.36)	0.359	
How would you rate your general health?	0.74 (0.49–1.12)	0.159	
Have you noticed any worsening of chronic conditions (e.g., asthma, heart disease) due to smog exposure?	0.04 (0.02–0.09)	0.001	
How often do you feel fatigued or unwell due to air pollution?	0.82 (0.54–1.24)	0.353	
**Asthma**		
On a scale of 1–5, how worried are you about the long‐term health effects of smog?	2.83 (1.67–4.80)	0.001	
How often do you feel stressed or anxious due to smog in your area?	6.21 (2.15–17.44)	0.001	
Have you experienced any changes in your mental well‐being (e.g., anxiety, depression) related to air pollution exposure?	0.74 (0.30–1.79)	0.508	
Do you feel that smog has negatively affected your quality of life?	3.29 (1.39–7.78)	0.001	
How has smog affected your daily activities (e.g., outdoor exercise, commuting)?	5.60 (2.52–12.44)	0.001	
How would you rate your general health?	1.35 (0.91–2.00)	0.127	
Have you noticed any worsening of chronic conditions (e.g., asthma, heart disease) due to smog exposure?	0.14 (0.07–0.31)	0.001	
How often do you feel fatigued or unwell due to air pollution?	0.58 (0.39–0.87)	0.001	
**Acute respiratory infection**		
On a scale of 1–5, how worried are you about the long‐term health effects of smog?	3.60 (2.11–6.12)	0.001	
How often do you feel stressed or anxious due to smog in your area?	9.45 (3.31–26.98)	0.001	
Have you experienced any changes in your mental well‐being (e.g., anxiety, depression) related to air pollution exposure?	6.20 (2.56–15.04)	0.001	
Do you feel that smog has negatively affected your quality of life?	2.05 (1.89–6.32)	0.001	
How has smog affected your daily activities (e.g., outdoor exercise, commuting)?	1.89 (0.84–4.22)	0.121	
How would you rate your general health?	1.57 (1.06–2.34)	0.024	
Have you noticed any worsening of chronic conditions (e.g., asthma, heart disease) due to smog exposure?	0.12 (0.05–0.24)	0.001	
How often do you feel fatigued or unwell due to air pollution?	0.66 (0.440.98)	0.042	
**Ischemic heart disease**		
On a scale of 1–5, how worried are you about the long‐term health effects of smog?	15.17 (8.46–27.17)	0.001	
How often do you feel stressed or anxious due to smog in your area?	25.11 (9.27–67.95)	0.001	
Have you experienced any changes in your mental well‐being (e.g., anxiety, depression) related to air pollution exposure?	0.74 (0.29–1.85)	0.001	
Do you feel that smog has negatively affected your quality of life?	5.82 (1.53–16.12)	0.525	
How has smog affected your daily activities (e.g., outdoor exercise, commuting)?	2.30 (0.99–5.32)	0.051	
How would you rate your general health?	0.32 (0.21–0.48)	0.001	
Have you noticed any worsening of chronic conditions (e.g., asthma, heart disease) due to smog exposure?	0.07 (0.03–0.16)	0.001	
How often do you feel fatigued or unwell due to air pollution?	1.21 (0.81–1.81)	0.346	

Abbreviations: CI = Confidence Interval; OR = Odds Ratio; SE = Standard error.

^a^ The reference category is: conjunctivitis;

^b^ This parameter is set to zero because it is redundant.

*p value < 0.05.

Concern about long‐term health effects is similarly significant for asthma outcomes, reflected by a coefficient of 1.04 (SE = 0.26), (OR = 2.83; 95% CI: 1.67–4.80; *p* = 0.001). There was a significantly stronger association between asthma and stress or anxiety related to smog, with a coefficient of 4.13 (standard error = 0.52), (odds ratio = 6.21; 95% confidence interval: 2.15–17.44; *p*‐value = 0.001). Respondents feeling that smog negatively impacts their quality of life exhibit significant associations with asthma outcomes; the coefficient is 2.97 (SE = 0.43), (OR = 3.29; 95% CI: 1.39–7.78; *p*‐value = 0.001). There was a significant negative association between worsening chronic conditions as a result of air pollution and asthma outcomes. This is demonstrated by a coefficient of −1.90 (standard error = 0.379), with an odds ratio of 0.14 (95% confidence interval: 0.07–0.31) and a *p* value of less than 0.001.

Concern about long‐term health effects is significantly associated with ARI outcomes; the coefficient is 1.28 (SE = 0.27), (OR = 3.60; 95% CI: 2.11–6.12; *p*‐value < 0.001). High levels of stress or anxiety related to smog show an even stronger correlation with ARI outcomes; this is reflected in a coefficient of 4.54 (SE = 0.53), (OR = 9.45; 95% CI: 3.31–26.98; *p*‐value < 0.001). Changes in mental well‐being related to air pollution exposure were significantly associated with ARI outcomes; the coefficient was positive at 1.82 (SE = 0.45), (OR = 6.20; 95% CI: 2.56–15.04; *p*‐value < 0.001).

Individuals expressing worry about long‐term health effects show a very strong association with IHD outcomes; as indicated by a coefficient of 2.71 (SE = 0.29), (OR = 15.17; 95% CI: 8.46–27.17; *p*‐value < 0.001). Anxiety or high levels of stress related to smog are exceedingly high for IHD; as shown by a coefficient of 3.22, (OR = 25.11, 95% CI: 9.27–67.95; *p*‐value < 0.001). Respondents feeling that smog negatively impacts their quality of life exhibit significant associations with asthma outcomes; the coefficient is 2.97 (SE = 0.43), (OR = 3.29; 95% CI: 1.39–7.78; *p*‐value = 0.001).

## 4 Discussion

Smog has a pervasive and multidimensional impact on both physical and mental health in the South Punjab, particularly on respiratory and ocular health outcomes. The findings demonstrated statistically significant correlations between smog exposure and various health outcomes, including COPD, ARI, asthma, IHD, and conjunctivitis. This discourse examines the consequences of these findings concerning the current literature, public health policies, and socio‐psychological impact of smog exposure.

Recent research has shown that air pollution is very poor for people's health all over South Asia. In this regard, one study found that people in Punjab cities that are prone to pollution had clusters of respiratory problems and conjunctivitis, which supports the spatial focus and results of our study [28]. Another study also talked about the two problems that smog exposure causes in urban Pakistan: bodily problems and mental problems. This is in line with what we found about the high levels of worry and stress. Urban green spaces can help reduce exposure to air pollution. However, our participants said that municipal policies weren't being enforced well and there wasn't much green space [29]. They stressed how important it was for the government to step in in Lahore, and our respondents' displeasure with local initiatives backed this up.

According to this study, the main health consequence of exposure to smog is respiratory illness. Patients with asthma or COPD are more likely to experience worse symptoms, particularly during smog events when the AQI is > 200. Persistent coughing, breathlessness, and elevated exposure to smog were strongly associated in multinomial logistic regression analysis.

Smog events are associated with ARI, which suggest that pollutants cause acute inflammatory reaction. Among the factors that contributed to this heightened risk was the lack of protective measures, such as the use of masks or air purifiers. There is an immediate need for focused interventions in rural areas because these problems are exacerbated by the limited accessibility of healthcare services. Although debates regarding air pollution tend to ignore ocular health, this study established strong associations between exposure to smog and symptoms pertaining to the eyes, including dryness, irritation, and conjunctivitis. These pollutants have an annoying effect on people's quality of life, which in turn causes them to use healthcare services more frequently. Controversial conjunctivitis outbreaks in southern Punjab during smog events call attention to the need for early intervention and education campaigns that promote the use of eye protection.

There are also concerns regarding the social and psychological effects associated with being exposed to smog. Individuals, particularly those residing in urban areas, are more likely to experience anxiety and stress after exposure to unhealthy air for an extended period of time. This is particularly true for rural residents. Owing to the weather, many locals have reported alterations to their typical commute patterns and a reduction in the amount of time they spend outside. These alterations in lifestyle, in conjunction with the anxieties associated with health, have a significant impact on mental health. In addition to the burden of fewer opportunities for recreation and increased responsibilities for caregiving, women and children frequently face additional challenges pertinent to their situation. To address these socio‐psychological challenges effectively, it is essential to incorporate mental health services into public health initiatives that aim to reduce pollution.

The purpose of this study is to investigate public sentiment regarding government programs designed to combat air pollution. This study's findings offer a unique perspective. In general, respondents expressed dissatisfaction with the effectiveness of these measures, despite the fact that some acknowledged the efforts that are currently being made. The individuals who participated in the discussion expressed their support for stricter regulations, particularly those that would improve public transportation systems and reduce emissions from industrial sources.

Several pieces of evidence suggest that awareness campaigns have the potential to significantly reduce the risk to health. Participants who were aware of how to protect themselves during smog events, such as wearing masks and staying indoors, reported fewer symptoms than those who were unaware of how to protect themselves. This highlights the importance of implementing education and outreach programs specifically targeted at promoting adaptive behaviors. Nevertheless, these campaigns continue to have a limited impact, particularly in rural areas, because of the lower literacy rates observed. Community radio and mobile health units are examples of innovative communication strategies that can be used to address this gap in the market.

The results of this study have several policy implications. The installation of real‐time air quality monitoring systems is an essential component of the stringent air quality regulations that are urgently required. Second, the government must prioritize the reduction of emissions from major sources, such as brick kilns, vehicle traffic, and agricultural practices. Reduce pollution levels significantly by promoting cleaner technologies and boosting sustainable agricultural practices. To combat the health impacts of pollution, it is necessary to upgrade the healthcare facilities in rural areas. Underprivileged communities may be able to obtain the medical attention they need more easily with the help of telemedicine and mobile health clinics. Additionally, healthcare providers could be better prepared to manage these conditions if the health effects of air pollution were included in the medical curriculum. Fourth, to tackle complex issues related to smog, it is essential for different sectors to work together. For effective strategies to be developed, there must be collaboration among healthcare providers, environmental scientists, lawmakers, and community leaders. Afforestation projects, public transportation upgrades, and other large‐scale endeavors may require public‐private partnerships to secure financing and conduct.

Expanding the scope of these studies to include more areas Pakistan would be beneficial. To better understand the effects of smog and to develop targeted solutions, comparative studies can reveal unique regional factors. Finally, studies on the link between air pollution and health might benefit from the application of cutting‐edge modeling approaches, such as machine learning.

This study demonstrates that air pollution is a problem not only in Pakistan but also in every other country in the world. There is a possibility that the lessons learned in the South Punjab could be used to inform methods in other low‐ and middle‐income countries that are coping with similar issues. To find a solution to this problem, which has repercussions in a variety of domains, international collaboration and information sharing will be required. The air Quality Guidelines from the World Health Organization and Sustainable Development Goals of the United Nations provide essential frameworks for directing initiatives of this nature.

### 4.1 Limitations and Future Aspects

Although this study had some limitations, it offered substantial conclusions. Due to the cross‐sectional design and possibility of bias in self‐reported data, causal conclusions cannot be drawn. Longitudinal studies should be the main focus of future research to assess the long‐term health effects of pollution and to identify causal relationships. A better understanding of individual susceptibility may also result from studying the genetic factors associated with pollution‐related diseases.

## 5 Conclusion

A major concern in the field of public health is the complex effects of pollution on the health and wellness of individuals, particularly in regions such as the South Punjab, where social, environmental, and healthcare concerns all come together. This study highlights serious eye health and respiratory issues caused by smog exposure, emphasizing the importance of targeted interventions. Integrating mental health services into public health programs is crucial, because of the socio‐psychological effects of smog. More strict air quality regulations, better healthcare infrastructure, and public awareness campaigns are important policy actions that can be implemented to lessen the impact of smog. Finding and executing workable solutions requires close cooperation among public organizations, healthcare providers, and members of the community. If people work together and share what they know, the world can move much more quickly to address air pollution and its effects on health. More need more comparative and longitudinal studies are needed to understand the long‐term impacts of pollution and to find solutions. Researchers, policymakers, and community members can all play a role in making smog‐affected areas better places to live in the future.

## Author Contributions

**Muhammad Muneeb Hassan:** conceptualization, investigation, writing – original draft, methodology, validation, visualization, writing – review and editing, software, formal analysis, project administration, data curation, supervision, resources. **Iqra Javaid:** writing – original draft, writing – review and editing, formal analysis, resources. **Farrukh Jamal:** project administration, formal analysis. **Muhammad Ameeq:** investigation, writing – original draft, writing – review and editing, software, formal analysis, project administration, data curation, resources. **Muhammad Danish:** resources, writing – review and editing. **Alpha kargbo:** data curation, supervision, resources, project administration, formal analysis, software, methodology, validation, visualization, funding acquisition, writing – original draft, investigation, conceptualization. **Ayesha Javed:** writing – review and editing, data curation.

## Ethics Statement

Ethical approval was obtained from the Institutional Review Board of DHQ Hospital Muzaffargarh No, 1003‐7/DHQ dated: 01 Jan 2025.

## Consent

This study was conducted in accordance with the **Declaration of Helsinki**. All participants were fully informed about the study's objectives, procedures, potential risks, and benefits. They were assured of confidentiality and their right to withdraw at any stage without any consequences. Verbal consent was obtained from participants due to **low illiteracy rates or cultural norms preventing written consent**. The **Institutional Review Board of DHQ Hospital Muzaffargarh** reviewed and approved the use of verbal consent, allowing thumb impressions to be used as documentation instead of electronic recording instruments, in compliance with institutional and ethical guidelines.

## Conflicts of Interest

The authors declare no conflicts of interest.

## Transparency Statement

The lead author affirms that this manuscript is an honest, accurate, and transparent account of the study being reported. No important aspects of the study have been omitted, and any discrepancies from the planned study design and methods have been clearly explained. All data were collected in accordance with ethical standards, and findings have been reported with integrity. The authors accept responsibility for the overall content of this article.

## Data Availability

The data that support the findings of this study are available on request from the corresponding author. The data are not publicly available due to privacy or ethical restrictions. The data supporting the findings of this study are available upon request from the first author, **Muhammad Muneeb Hassan.** The data are not publicly available because of restrictions on their information, which could compromise the privacy of the research participants. The corresponding author can access the data, models, and codes that support the findings of this study.
